# Interaction of light regimes and circadian clocks modulate timing of pre-adult developmental events in *Drosophila*

**DOI:** 10.1186/1471-213X-14-19

**Published:** 2014-05-16

**Authors:** Pankaj Yadav, Madhumohan Thandapani, Vijay Kumar Sharma

**Affiliations:** 1 Chronobiology Laboratory, Evolutionary and Organismal Biology Unit, Jawaharlal Nehru Centre for Advanced Scientific Research, P. O. Jakkur, Bangalore, Karnataka 560064, India

**Keywords:** Circadian clocks, Faster development, Light regimes, Selection, Development time, Pre-adult stages

## Abstract

**Background:**

Circadian clocks have been postulated to regulate development time in several species of insects including fruit flies *Drosophila melanogaster*. Previously we have reported that selection for faster pre-adult development reduces development time (by ~19 h or ~11%) and clock period (by ~0.5 h), suggesting a role of circadian clocks in the regulation of development time in *D. melanogaster*. We reasoned that these faster developing flies could serve as a model to study stage-specific interaction of circadian clocks and developmental events with the environmental light/dark (LD) conditions. We assayed the duration of three pre-adult stages in the faster developing (FD) and control (BD) populations under a variety of light regimes that are known to modulate circadian clocks and pre-adult development time of *Drosophila* to examine the role of circadian clocks in the timing of pre-adult developmental stages.

**Results:**

We find that the duration of pre-adult stages was shorter under constant light (LL) and short period light (L)/dark (D) cycles (L:D = 10:10 h; *T20*) compared to the standard 24 h day (L:D = 12:12 h; *T24*), long LD cycles (L:D = 14:14 h; *T28*) and constant darkness (DD). The difference in the duration of pre-adult stages between the FD and BD populations was significantly smaller under the three LD cycles and LL compared to DD, possibly due to the fact that clocks of both FD and BD flies are driven at the same pace in the three LD regimes owing to circadian entrainment, or are rendered dysfunctional under LL.

**Conclusions:**

These results suggest that interaction between light regimes and circadian clocks regulate the duration of pre-adult developmental stages in fruit flies *D. melanogaster*.

## Background

Most holometabolous insects including fruit flies *D. melanogaster* go through three discrete developmental stages namely egg, larvae and pupae. At an ambient temperature of 25°C, eggs typically take 18–24 h to hatch followed by the larval stage which spans for ~4 days, during which developing larvae pass through three instars [1]. The pupal stage starts after the third instar larval stage, lasts for another 4 days, subsequently leading to wing-pigmentation followed by adult emergence, hence the entire pre-adult developmental duration of *Drosophila* spans ~9 days. Wing-pigmentation is considered to be the last stage of the fly development and therefore, no further major change in pupa is expected to take place thereafter [2-4].

Circadian clocks have been implicated in the temporal regulation of pre-adult development in *D. melanogaster*[5,6] as several studies have reported that the pre-adult development time and clock period show positive correlation [7-11]. Furthermore, studies on insects including *Drosophila* have reported rhythmicity in several developmental events such as egg-hatching [12,13], pupation [14,15] and wing-pigmentation [2,16], implying a role of circadian clocks in timing pre-adult stages.

While adult emergence in insects including *Drosophila* is under the control of circadian clocks [17-19], evidence suggests that clocks begin ticking in the fly as early as the third instar larval stage and is functional for the most part of pre-adult development [20-23]. Additionally, *Drosophila* larvae are known to show rhythmicity in light avoidance behaviour [24], which is probably the earliest and the only clock-driven pre-adult behaviour reported thus far in *Drosophila*. Light/dark (LD) cycles are known to be one of the strongest zeitgebers for the adult emergence rhythm of fruit flies and is known to play a key role in entraining (synchronising) the circadian clocks present during early developmental stages [25,26]. Adult emergence rhythm in *Drosophila* is known to be entrained by a wide range of LD cycles [8,19], causing a significant impact on its pre-adult developmental duration; speeding it up under constant light (LL), relative to 12:12 h LD cycles and slowing it down under DD [8,27,28]. In addition, pre-adult development time in *Drosophila* is reported to be positively correlated with the period of LD cycles (*T* cycles), suggesting a role of period of circadian rhythms and/or LD cycles in the regulation of pre-adult developmental duration [8,29]. While it is known that the duration of pre-adult development would be greatly affected by environmental LD cycles [8,27,28], which of the developmental stages would be affected the most is still unknown.

Previously we had reported a corollary to the above - that populations of *D. melanogaster* subjected to selection for faster pre-adult development under DD, evolve shorter development time (~29 h shorter than the controls, after 50 generations of selection) and circadian clocks with free-running period ~0.5 h shorter than the controls [11]. Additionally, we had reported that speeding up of development in these populations is achieved by concurrent reduction in the duration of almost all pre-adult stages [11]. Evolution of faster running circadian clocks in the faster developing (FD) flies suggests a link between circadian clocks and development time, similar to what has been implied in several previous studies [7-10,30-32]. Considering the ability of circadian clocks to entrain to a wide range of LD cycles, and a sizable difference in development time between the faster developing and control populations [8], in the present study, we decided to examine the effects of interaction between circadian clocks and light regimes in the timing of pre-adult developmental stages by assaying the duration of several pre-adult stages in the selected Faster Developing (FD) and control Baseline Developing (BD) populations under three light/dark (LD) cycles [LD 10:10 h (*T20*), LD 12:12 h (*T24*) and LD 14:14 h (*T28*)] and two constant conditions (constant light - LL and constant dark - DD).

## Results

### Egg-hatching time assay

**Figure 1:**
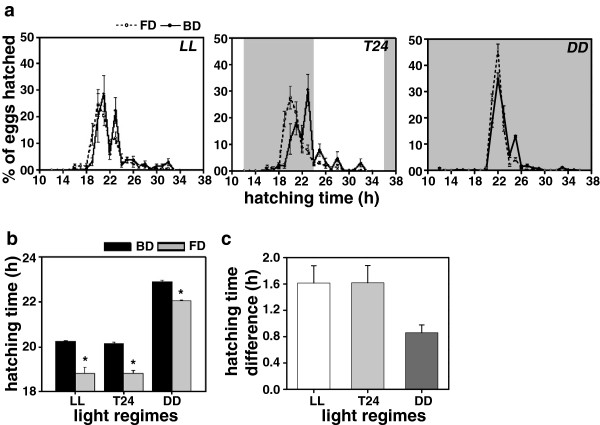
**Pupation-time under LL, *T20, T24, T28 *and DD. (a)** Waveforms showing patterns of pupation-time of the selected (FD) and control (BD) flies under constant light (LL), 10:10 h light/dark (LD) cycles (*T20*), 12:12 h LD cycles (*T24*), 14:14 h LD cycles (*T28*) and constant dark (DD) conditions. **(b)** The pupation-time (time interval from egg-to-pupae formation) of the FD and BD flies under LL, *T20*, *T24*, *T28* and DD, showing effects of light regimes. **(c)** Difference between the pupation-time of the FD and BD flies under LL, *T20*, *T24*, *T28* and DD conditions, showing light regime effect on the egg-to-pupation duration. All other details are same as in Figure 1.

Since egg-hatching in *Drosophila* typically lasts for 18 to 24 h and it would require a very high resolution in data to pick-up any difference between the selected and control flies, we estimated this duration only in LL, *T24* and DD conditions. Under all the three light regimes, the egg-hatching waveform of the FD flies was shifted earlier compared to the BD controls (Figure 1a), and the average duration of the egg-stage was shorter in the FD flies compared to the BD controls by ~1.6 h under LL, ~1.6 h in *T24* and ~0.9 h in DD (Figure 1b). ANOVA revealed a statistically significant effect of light regime (L), stock (S); however, the effect of L × S interaction was statistically not significant (Figure 1b, c; Table 1). Under all the three light regimes, egg-hatching time of the FD flies was significantly shorter than the BD controls (Figure 1b; Table 1). However, the difference in the egg-hatching time between the FD and BD flies did not differ statistically between the three environmental conditions (LL - 1.61 h; *T24* - 1.62 h; and DD - 0.86 h; Figure 1c; Table 2). Shortening of egg-hatching duration in the FD flies, under all the three light regimes, indicates that response to selection for faster development overrides the effects of light regimes (Figure 1c).

**Table 1:** Results of ANOVA on data from different assays

**Effect**	***df***	**MS effect**	***df *** **error**	**MS error**	***F***	***p*-level**
***Egg-hatching time assay under LL, T24 and DD***						
Light regime (L)	2	23.67	6	0.23	104.21	0.0001
Stock (S)	1	8.8	3	0.71	12.38	0.04
L × S	2	0.19	6	0.15	1.29	0.34
***Pupation-time assay under LL, T20, T24, T28 and DD***						
Light regime (L)	4	275.06	12	5.88	46.81	0.0001
Stock (S)	1	858.22	3	4.03	212.73	0.0007
L × S	4	8.02	12	1.89	4.25	0.02
***Wing-pigmentation time assay under LL, T20, T24, T28 and DD***						
Light regime (L)	4	419.06	12	2.41	173.58	0.0001
Stock (S)	1	1831.77	3	2.41	760.71	0.0001
L × S	4	16.27	12	1.48	10.99	0.0006
***Egg-to-adult development time assay under LL, T20, T24, T28 and DD***						
Light regime (L)	4	475.81	12	7.15	66.59	0.0001
Stock (S)	1	2279.14	3	0. 71	3219.08	0.0001
L × S	4	8.36	12	2.41	3.48	0.04

**Table 2:** Results of ANOVA on difference between stocks at various developmental stages

**Effect**	***df***	**MS effect**	***df *** **error**	**MS error**	***F***	***p*-level**
***Egg-hatching time difference***						
Light regime (L)	2	0.38	6	0.29	1.29	0.34
***Pupation-time difference***						
Light regime (L)	4	16.03	12	3.78	4.25	0.02
***Wing-pigmentation time difference***						
Light regime (L)	4	32.54	12	2.96	10.99	0.0006
***Egg-to-adult development time difference***						
Light regime (L)	4	16.71	12	4.48	3.48	0.04

### Pupation and wing-pigmentation time assays

**Figure 2:**
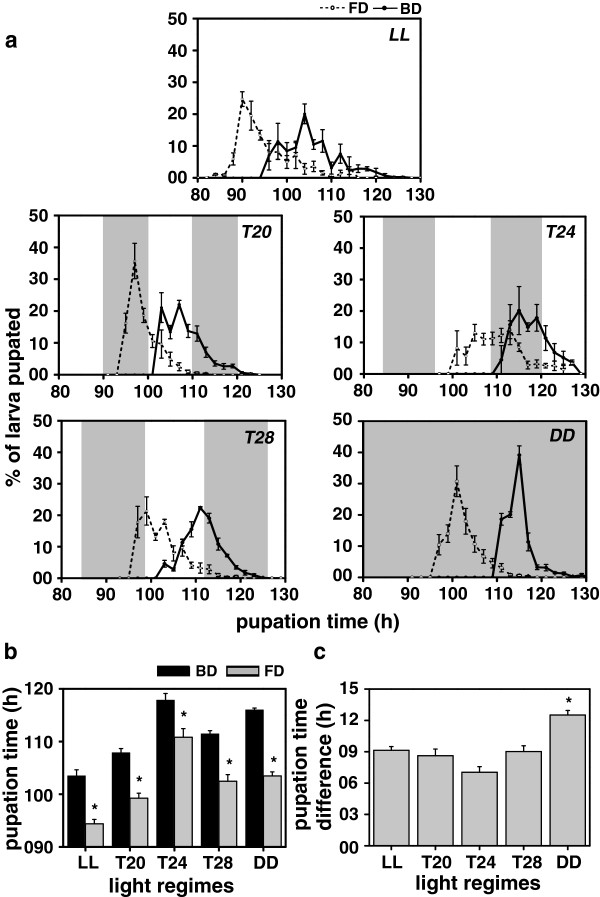
**Pupation-time under LL, *T20, T24*, *T28 *and DD. (a)** Waveforms showing patterns of pupation-time of selected (FD) and control (BD) flies under constant light (LL), 10:10 h light/dark (LD) cycles (*T20*), 12:12 h LD cycles (*T24*), 14:14 h LD cycles (*T28*) and constant dark (DD) conditions. **(b)** The pupation-time (time interval from egg-to-pupae formation) of the FD and BD flies under LL, *T20*, *T24*, *T28* and DD, showing the effect of light regimes. **(c)** Difference between the pupation-time of the FD and BD flies under LL, *T20*, *T24*, *T28* and DD conditions, showing light regime effect on the egg-to-pupation duration. All other details are same as in Figure 1.

**Figure 3:**
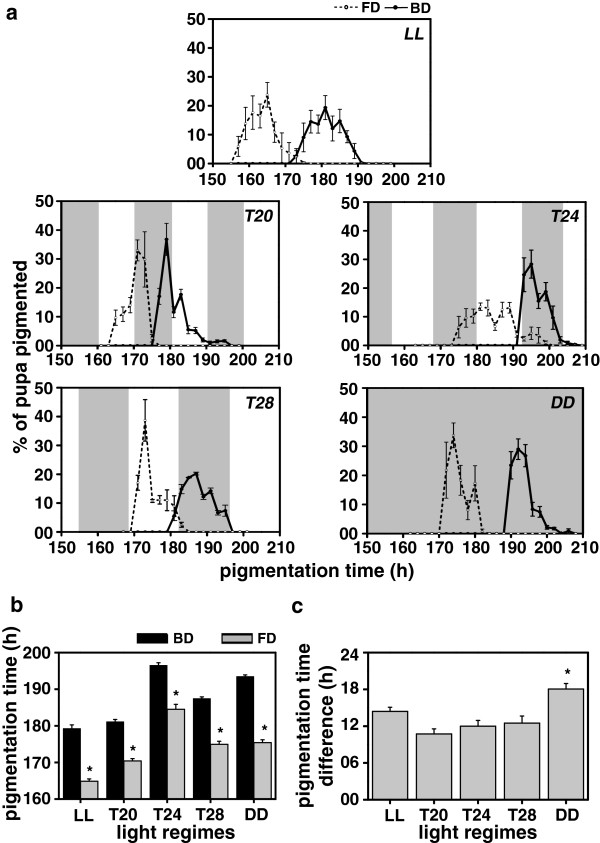
**Wing-pigmentation-time under LL, *T20, T24*, *T28 *and DD. (a)** Waveforms showing differences in wing-pigmentation time of the selected (FD) and control (BD) flies stocks under constant light (LL), 10:10 h light/dark (LD) cycles (*T20*), 12:12 h LD cycles (*T24*), 14:14 h LD cycles (*T28*) and constant dark (DD) conditions. **(b)** The wing-pigmentation time (time interval from egg-to-wing-pigmentation) of the FD and BD flies under LL, *T20*, *T24*, *T28* and DD conditions. **(c)** Difference between the wing-pigmentation time of the FD and BD flies assayed under LL, *T20*, *T24*, *T28* and DD conditions, showing light regime effect on egg-to-wing-pigmentation duration. All other details are same as in Figure 1.

These assays were performed under five different regimes (LL, *T20*, *T24*, *T28* and DD). Under all the five assay light regimes, pupation (Figure 2) and wing-pigmentation time (Figure 3) of the FD flies was significantly shorter than the BD controls. The pupation-time difference between the FD and BD flies under LL, *T20*, *T24*, *T28* and DD conditions was about 9, 8.6, 7, 9 and 12.5 h respectively (Figure 2b, c). Under *T20*, the FD flies pupated mostly in the dark and the BD flies in the light, while in *T28*, flies from both the stocks pupated during the light phase. However, under *T24*, pupation of the FD flies spanned over both dark and light phases, while that of the BD flies was mostly restricted to the dark phase and partly to the light phase. The FD flies pupated earlier than the BD controls under both LL and DD (Figure 2a). ANOVA on the pupation-time data revealed a statistically significant effect of L, S and L × S interaction (Figure 2b, c; Table 1). Post-hoc multiple comparisons using Tukey’s test revealed that under all the five light regimes, pupation-time of the FD flies was significantly shorter compared to the BD controls. Pupation-time of both the stocks was shorter under LL followed by *T20* and *T28*, while it was longer in *T24* and DD (Figure 2b). ANOVA on the pupation-time difference (BD-FD) data revealed a statistically significant effect of L (Figure 2c; Table 2) with the difference being smaller under LL, *T20*, *T24* and *T28*, compared to DD. Thus, under entraining as well as rhythm-abolishing conditions, clock-mediated difference in pupation-time between the FD and BD flies is significantly reduced, while under free-running condition, the difference persisted. These results suggest that circadian clocks regulate pupation-time in *Drosophila*.

The difference in wing-pigmentation time between the FD and BD stocks was about 14 h in LL, 11 h in *T20*, 12 h in *T24*, 12.5 h in *T28* and 18 h in DD (Figure 3). Under *T20*, wing-pigmentation of the FD flies started in the light phase and was completed in the dark phase, while in the BD flies it started in the dark and ended during the light phase. In *T28*, wing-pigmentation of the FD flies was confined to the light phase, while that of BD flies was confined to the dark phase (Figure 3a). Under *T24*, pigmentation occurred during both light and dark phases in the FD flies, while in the BD flies it occurred only during the dark phase, and under both LL or DD, the FD flies pigmented earlier than the BD controls (Figure 3a). ANOVA on the wing-pigmentation time data revealed a statistically significant effect of L, S and L × S interaction (Figure 3b, c; Table 1). Post-hoc multiple comparisons using Tukey’s test revealed that under all five light regimes, wing-pigmentation time of the FD flies was significantly shorter than the BD controls, and the mean wing-pigmentation time of both the populations was shortest under LL, followed by *T20* and *T28*, while it was longest in *T24* and DD (Figure 3b). The wing-pigmentation time difference between the two stocks was significantly shorter under LL, *T20, T24* and *T28* compared to DD (Figure 3c). ANOVA on the difference data revealed a statistically significant effect of L (Figure 3c; Table 2), indicating that under LL and entrained conditions (*T20, T24* and *T28*), clock-mediated difference in pigmentation-time between the FD and BD flies is significantly reduced while it persists in free-running condition (DD). This suggests that circadian clocks regulate timing of wing-pigmentation in *Drosophila.*

### Egg-to-adult development time

**Figure 4:**
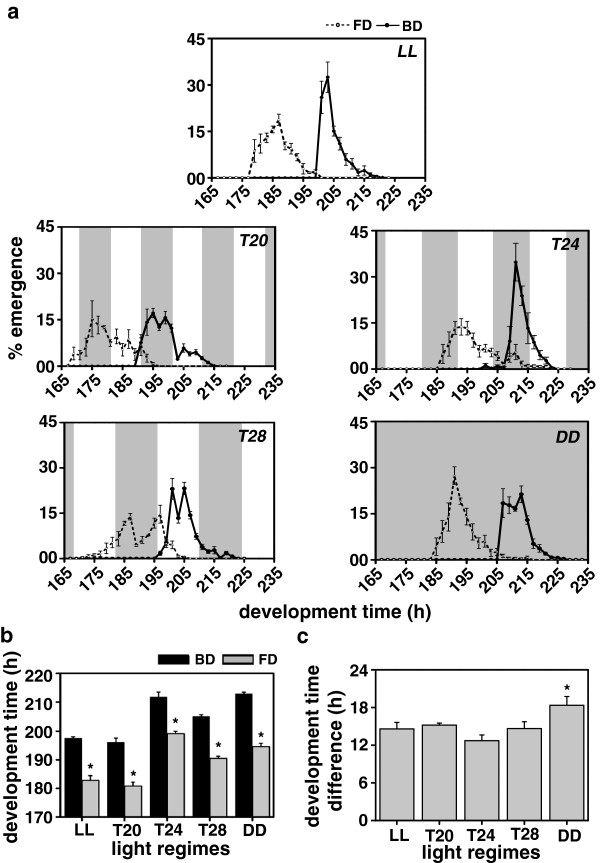
**Egg-to-adult development time under LL, *T20, T24, T28 *and DD. (a)** Waveforms showing difference in pre-adult development time of the selected (FD) and control (BD) flies under constant light (LL), 10:10 h light/dark (LD) cycles (*T20*), 12:12 h LD cycles (*T24*), 14:14 h LD cycles (*T28*) and constant dark (DD) conditions. **(b)** The pre-adult development time (time interval from egg-to-adult emergence) of the FD and BD flies under LL, *T20*, *T24*, *T28* and DD conditions. **(c)** Difference between the pre-adult development time of the FD and BD flies under LL, *T20*, *T24*, *T28* and DD conditions showing light regime effect on the egg-to-adult emergence duration. All other details are same as in Figure 1.

After 40 generations of selection, the difference in pre-adult development time between the FD and BD stocks was about 15 h in LL, 15 h in *T20*, 13 h in *T24*, 15 h in *T28* and 18 h in DD (Figure 4). Pre-adult emergence profiles of the FD and BD flies showed similar pattern across all the light regimes, with the FD flies emerging consistently earlier than the BD controls (Figure 4a). However, difference in development time between the two populations under LL and three LD cycles was significantly smaller than that in DD (Figure 4b, c). Under *T20*, both FD and BD flies emerged in the dark and emergence ended in the light phase of the next cycle, and in *T24,* emergence of the FD flies was confined to the light phase, while that of the BD flies to the dark phase (Figure 4a). Under *T*28, the FD flies started emerging in the middle of the light phase, continued emerging in the dark phase and their emergence was completed only in the light phase of the next cycle, while that of the BD flies was confined only to the light phase (Figure 4a). ANOVA revealed a statistically significant effect of L, S and L × S interaction (Figure 4b; Table 1). Post-hoc multiple comparisons using Tukey’s test revealed that under all the five light regimes, egg-to-adult development time of the FD flies was significantly shorter than the BD controls, and the mean development time of both the populations was shortest under LL followed by *T20* and *T28*, and was longest under *T24* and DD (Figure 4b). ANOVA on the difference data revealed that the effect of L was statistically significant (Figure 4c; Table 2). The difference in development time (BD-FD) between the selected and control flies under the entraining conditions (*T20*, *T24* and *T28*) and rhythm abolishing condition (LL) was significantly reduced compared to that under free-running condition (DD). These results suggest that circadian clocks regulate the duration of pre-adult development in *D. melanogaster*.

### Free-running period of adult emergence and activity/rest rhythms

We estimated the period of adult emergence and activity/rest rhythms from the emergence and activity data collected after 40 generations of selection (Additional file 1: Figure S1). The FD populations had a significantly shorter (F_*1,3*_ = 12.78; *p* < 0.037; Additional file 1: Figure S1) period of adult emergence rhythm (23.6 ± 0.19 h mean ± SEM) compared to the BD controls (24.4 ± 0.06 h). Similarly, the FD populations had a significantly shorter (F_*1,3*_ = 66.82; *p* < 0.004; Additional file 1: Figure S1) period of activity/rest rhythm (23.9 ± 0.02 h) compared to the BD controls (24.3 ± 0.06 h). These results suggest that selection for faster pre-adult development results in a correlated shortening of clock period in the FD populations compared to the controls.

## Discussion

Our previous study had shown that, under DD, developmental events such as egg-hatching, pupation and wing-pigmentation occur earlier in the FD flies than in the BD controls [11]. We hypothesized that if the duration of pre-adult stages is entirely clock-mediated, the difference in the duration between the FD and BD flies would disappear (a) under the three LD cycles, due to the fact that circadian clocks of flies from both the stocks would run at the same pace owing to circadian entrainment, and (b) under LL, wherein their clocks would be rendered dysfunctional. Indeed, the results of our study revealed that the difference in the duration of pre-adult stages between the FD and BD stocks was significantly reduced under the three LD cycles and LL compared to DD. However, exposure to LD or LL regimes did not eliminate the difference in the duration of pre-adult developmental stages between the FD and BD flies completely, which suggests that circadian clocks only partly regulate the timing of pre-adult developmental stages in *D. melanogaster*.

Exposure to light (LL and LD) significantly reduces the egg-hatching time but has no effect on the difference between the FD and BD stocks, suggesting that egg-hatching is light-mediated but a clock-independent process. In the current study we have estimated the time of egg-hatching with reference to the egg-stage (emergence of the first instar larvae), which is likely to represent the total duration of egg-stage. Our results are also consistent with the findings of previous studies which reported that a single light pulse administered immediately after the egg-hatching stage is sufficient to entrain the circadian clocks of *Drosophila* while the clocks start ticking only at the third instar stage [20-23].

Earlier studies have shown that pupation is gated in mosquitoes such as *Anopheles gambiae*[33] and *Aedes taeniorhynchus*[34,35], and the *period* (*per*) mRNA expression during pupation in fruit flies *D. melanogaster* is under circadian clock control [3]. Moreover, several developmental events such as egg-hatching [12,13], pupation [14,15] and wing-pigmentation [2,16], have been reported to show rhythmicity, which implies that circadian clocks are likely to interact with several gated events during the egg-hatching, pupation and wing-pigmentation stages. Such gated events and the transition time from one stage to another is likely to create constraints on the developmental rates, which in turn would cause reduction in the proportionate differences in the duration of pupal (Figure 2b), wing-pigmentation (Figure 3b) and pre-adult developmental stages of the FD and BD flies (Figure 4b). Concurrently, difference in the duration of the two pre-adult stages between the FD and BD stocks was found to be greater under DD compared to the three LD or LL conditions (Figures 2c and 3c). This could partly be due to the fact that the difference in clock period between the FD and BD stocks persists under DD, while it disappears under LD and LL conditions.

Under all the five light regimes, pupation, wing-pigmentation and adult emergence in the control populations began only after these processes were nearly completed in the selected populations (Figures 2a, 3a and 4a). This indicates that the impact of selection for faster pre-adult development is much stronger than that of light-regimes; however, difference in the duration of pre-adult stages between the FD and BD flies varied between the entraining, rhythm abolishing and free-running light regimes (Figures 2 and 3). This suggests that time-to-pupation, wing-pigmentation and adult emergence is a function of the period of circadian clocks, implying that interaction of light regimes and circadian clocks is a key determinant for the timing of pre-adult developmental events in *Drosophila*.

In insects, timing of ecdysone (a steroid hormone) release is known to trigger pupal development [36,37]; its premature release speeds-up development while delayed release slows it down [38,39]. In hornworms *Manduca sexta*, opening of gate during the larval stage is believed to be the signal that regulates the timing of release of prothoracicotropic hormone [39]. Thus, it is suggested that modulation of pre-adult development time may be due to the altered timing of prothoracicotropic hormone release which is primarily caused by the altered timing and the duration of gate opening at different developmental stages. Although in our present study we did not estimate ecdysone levels, the observation that clocks of both selected and control flies appear to have entrained to all the imposed LD cycles at egg (Figure 1a), larval (Figure 2a) and pupal (Figure 3a) stages provides an indirect evidence for the difference in the timing of ecdysone release being regulated by the gating of various developmental stages.

The timing of adult emergence in *Drosophila* depends upon a number of factors including developmental states, phase and period of circadian rhythms and on the external environmental conditions [4,7,8,28]. LD cycles restrict emergence of adults to a narrow window of time called as “allowed zone” or “gate” of emergence [4,17,19,25,40]. Since circadian rhythms in *Drosophila* are abolished under LL, gating of emergence is likely to be absent in this condition, and therefore, developing individuals would enter the subsequent developmental stages without any delay, thereby speeding up the pre-adult developmental stages [28]. On the other hand under LD cycles, duration of developmental events is likely to be determined by an interaction between the developmental states and circadian gating created by the LD cycles, which is likely to be altered depending on the length of the LD cycles and the timing of light/dark phases [4,8]. Under DD, where circadian clocks free-run, development time would be determined by some interactions between the developmental states and circadian clocks, and thus development time of flies in this regimes would be comparable to that in *T24* (Figure 4b). Therefore, timing of pre-adult developmental events in *Drosophila* is expected to follow the trend of being fastest under LL < *T20* < *T24* or DD < *T28*. The results of our present study are consistent with these trends and with earlier findings as it shows that the mean development time of the two major developmental stages is shorter under LL and *T20* and longer in *T24*, *T28* and DD [8,27]. As expected, flies take similar amount of time to develop under *T24* and DD (Figure 4b). Interestingly, lack of emergence gating under LL does not supersede the extent of shortening of development under *T20*. Among the LD cycles, development time was shortest under *T20*, followed by *T24* and *T28*, with flies taking shorter time to develop under *T28* compared to *T24*. This hints at the possibility of a threshold, beyond which the pre-adult development of *Drosophila* cannot be slowed down any further, at least not by light, thus constraining the nature of correlation between development time and the period of light regime beyond a particular limit.

Interestingly, the timing of developmental events in the selected as well as control populations which accompanied small but statistically significant difference in clock period (~0.5 h) was considerably affected by light regimes; the difference in the duration of developmental stages is shortened under LL and the three LD cycles compared to DD. However, irrespective of the state of temporal organization, flies selected to develop faster as pre-adults had shorter egg-to-adult developmental duration compared to the controls, under the three LD cycles and LL conditions. This suggests that to a large extent, the difference in development time between the selected and control flies is independent of the difference in their clock period. Therefore, development time differences between the two stocks under different light regimes alone cannot be taken as evidence to suggest the role of circadian clocks in the regulation of the timing of pre-adult developmental stages in *Drosophila*.

Since clock independent and light mediated pathways are also likely to play some role in the regulation of development time, genetic experiments involving flies with modified circadian clocks would be helpful. However, mutant lines are often inbred, which may yield spurious genetic correlations between fitness components [41]. Therefore, in such studies, use of mutant flies may not be an ideal choice [42], and the best strategy would be to examine such correlations in natural populations, where sufficient variation in clock period and development time is likely to exists. The other alternative would be to examine such correlations in large replicate populations, selected for different clock period values. Several studies have shown that most insect species are sensitive to light especially in the blue-green region of spectra (400 to 500 nm), while a few species show sensitivity up to the red end of the spectrum [19]. Moreover, a study in which eggs of pink bollworm *Pectinophora gossypielia* were exposed to monochromatic light after the midpoint of embryogenesis, showed initiation of the egg-hatching rhythm optimally between 390 and 480 nm and a sharp cut-off above 520 nm [43]. Similarly, larvae of the cabbage white butterfly *Pieris brassicae* were found to be more sensitive to wavelengths between 400 to 520 nm while insensitive to red light (above 580 nm)[44]. In a previous study, it was reported that *Drosophila* larvae are highly sensitive to 500 nm (green), 420 nm (violet) and 380 nm (ultraviolet) [45]. Since the spectral peaks of the light source used in our study correspond to 570 nm (green) and 420 nm (violet), it is likely that the light input pathways affecting the duration of pre-adult stages comprise of photopigments sensitive to 570 and 420 nm (Additional file 1: Figure S2). Moreover, two additional peaks in the spectrum corresponding to 570–590 nm (yellow-orange), 620 nm (red) suggests the likelihood of these wavelengths also influencing the developmental rates of fruit flies.

The faster developing (FD) populations have evolved small but consistent (across 75 generations) and reproducibly (across four replicate populations) shorter free-running period of circadian adult emergence and activity/rest rhythm (by ~0.5 h) compared to the controls ([11]; Additional file 1: Figure S1). However, it is arguable whether such changes are really significant because it is easy to obtain a small period change without substantial impact on the core clock mechanism. There are numerous examples in circadian biology literature where differences in behavioural rhythms are not found to be correlated with the molecular or neural mechanisms. For instance, differences in the activity/rest rhythm seen in fruit fly populations maintained under semi-natural conditions did not correlate with changes at the molecular or neural levels [46]. Similarly difference in activity/rest rhythm of different *Drosophila* species was found to occur in spite of very little difference at the molecular and neural levels [47,48]. The fact that all the four populations of the faster developing flies underwent changes in the same direction (BD1-FD1 = 0.44 h; BD2-FD2 = 0.71 h; BD3-FD3 = 0.41 h; BD4-FD4 = 0.52 h; [11]), suggests that the period differences are not because of random genetic drift but due to the imposed selection for faster pre-adult development. Multigenic traits such as clock period showing a consistent and reproducible change is a testament to adaptive evolution of circadian clocks as a correlated response to selection for faster development. Furthermore, it is unlikely that the genetic architecture underlying the circadian phenotype would permit large changes in circadian period because of multigenic control. Our results are also consistent with the notion that while direct response to selection on a trait is limited to few times (four to six) the standard deviation of the mean trait value, correlated responses are much smaller ([49,50]; references therein).

In summary, across successive stages of development, circadian clocks of *Drosophila* under various light regimes interact with several “gated” developmental events. Provided development is a light regime-mediated clock-controlled event, stage-specific clock or light regime-dependent effects can only be observed by a manipulation in the clock speed which can then be detected in terms of relative difference in development time across the light regimes. Our study reveals that unlike the egg stage, most pre-adult developmental stages in fruit flies *D. melanogaster* are light sensitive and clock-controlled.

## Conclusions

We found that several pre-adult developmental stages of *D. melanogaster* are susceptible to light and its duration is determined by the interaction between developmental clocks and circadian gating created by LD cycles, suggesting that interaction of light regimes and circadian clocks modulate the timing of pre-adult events in *Drosophila.*

## Methods

### Experimental populations

We used four replicates each of large, outbred laboratory populations of *D. melanogaster* (*N* ~ 1200, with roughly equal number of males and females), namely the FD_*1–4*_ (Faster Developing - selected) and BD_*1–4*_ (Baseline Developing - controls). The baseline populations were derived from four outbred LL_*1–4*_ populations [51], and maintained for ~100 generations, at moderate larval and adult densities (~60 per vial), under DD with constant temperature (25 ± 0.5°C) and relative humidity (~75%), on a 21 day discrete generation cycle. Thus, the BD populations were maintained under DD for ~100 generations prior to initiating four additional faster developing FD populations. The FD_*1*_ population was derived from BD_*1*_, FD_*2*_ from BD_*2*_, FD_*3*_ from BD_*3*_ and FD_*4*_ from BD_*4*_ populations. Thus, each of the FD populations was derived from its corresponding BD population and therefore, the selected and control populations bearing identical numerical subscripts are genetically more closely related to each other than the populations with which they share the selection regime. Temperature (~25.0°C) and relative humidity (~75%) were monitored continuously using Quartz Precision Thermo-Hygrograph, Isuzu Seisakusho Co, LTD and were found to be stable throughout the study.

Each replicate population was separately maintained in a plexi-glass cage (25 × 20 × 15 cm^3^) supplemented with banana-jaggery food medium (henceforth, banana medium). To start a new generation, adult flies were provided with banana medium supplemented with live yeast paste in a petri dish for 2 days and from these petri dishes 60–80 eggs were dispensed into glass vials (9 cm height × 2.4 cm diameter). For the BD stocks, adult flies emerging from 24 such vials containing 6 ml of banana medium were transferred into plexi-glass cages on the 12^th^ day after egg-collection. For the FD stocks, 80 such vials containing 6 ml of banana medium were used for each population and from each vial the first 15–20 emerging flies (approximately 25% faster) were collected and transferred into plexi-glass cages on the day of emergence. For starting a new generation, the next sets of eggs were collected 21 days after the previous egg-collection date.

To eliminate any possible non-genetic effect of parental rearing condition between the FD and BD stocks, before the initiation of every assay, all the eight populations were subjected to a common rearing condition (BD-type) for one generation. For this, 50–60 eggs laid over 12 h on banana food in the running cultures of each of the FD_*1–4*_ and BD_*1–4*_ populations were dispensed into 24 glass vials containing 6 ml of banana medium. These vials were kept under DD until all the adult flies emerged and 3 days later these flies were transferred into plexi-glass cages. These caged populations will be referred to as “standardized populations”. From these standardized populations, eggs laid for 2 h on banana medium were collected for the assays. All assays were performed at the 40^th^ generation of selection and development time of different pre-adult stages were measured with reference to the egg stage, hence are referred in the text as egg-to-hatching, egg-to-pupation, egg-to-wing-pigmentation and egg-to-adult emergence duration.

### Egg-hatching time assay

Eggs of approximately identical age were collected from the standardized populations by placing a fresh food plate in the population cage for 1 h. The plate was then replaced by another fresh food plate for the next 1 h. Thus, antecedent eggs retained in the female body were avoided in all the assays. Eggs were collected from the food plate and dispensed on 0.5 cm^2^ agar pieces with exactly 30 eggs arranged in 5 rows and 6 columns and placed in petri dishes for the ease of observation of the egg-hatching process. During the egg-collection, eggs were moistened every 4–5 min with few drops of water to prevent them from drying. Since *D. melanogaster* eggs start hatching 18–24 h after being laid and all eggs hatch in a span of few hours, we counted the number of eggs that hatched every 1 h, starting 12 h after egg-collection. The egg-hatching time assay was done under LL, *T24* and DD conditions. From this data, egg-hatching time was estimated as the time interval between the mid-point of 1 h egg-collection window and of the 1 h assay duration during which the egg hatched. Since each FD population was derived from its respective BD population, egg-hatching time difference was calculated by subtracting hatching time of FD_*1*_ from BD_*1*_, similarly egg-hatching time of FD_*2*_, FD_*3*_ and FD_*4*_ population was subtracted from that of BD_*2*_, BD_*3*_ and BD_*4*_ populations respectively. This was also applied for the pupation, wing-pigmentation and pre-adult development time difference estimations.

### Pupation and wing-pigmentation time assays

In order to assess the role of circadian clocks in the temporal regulation of two major pre-adult stages (larval and pupal), we performed three separate experiments namely the pupation-time (duration from egg-to-termination of third instar larva), wing-pigmentation time (duration from egg-to-wing pigmentation) and pre-adult development time assays (duration from egg-to-adult emergence). Starting the third day after egg-collection, vials were continuously monitored for pupae (when third instar larva encapsulates inside a hard and dark colored puparium) every 2 h. In each regime, 30 eggs were placed in each glass vial containing 6 ml of banana medium and 10 such vials were used for each population. The number of larvae that pupated in each vial were scored and marked with a circle on the glass vial. These 2 hourly checks were continued until no new pupae were formed for 2 consecutive days. The pupae undergo pigmentation due to wing development, so we continued with the same experimental set-up for the wing-pigmentation assay (blackening of a mature pupa, which is considered as the complete maturation of pre-adult development). The wing-pigmentation time was recorded every 2 h by marking a cross sign on the encircled mature pupae already marked during the pupation-time assay.

### Egg-to-adult development time assay

For the pre-adult development time assay, flies from the standardized FD_*1–4*_ and BD_*1–4*_ stocks were allowed to lay eggs on banana medium. In order to increase the egg-laying capacity of flies, 2 days prior to egg-collection live yeast paste was supplemented on the banana medium. Flies were allowed to lay eggs for 2 h and exactly 30 eggs were collected and dispensed into glass vial containing 10 ml of banana medium. Ten vials for each replicate population were introduced into five light regimes (LL, *T20*, *T24*, *T28* and DD). Thus, a total of 400 vials were used for this assay (10 vials × 8 populations × 5 light regimes). Eggs were collected under microscope with the help of a moistened ‘000’ size brush and introduced into different light regimes. For assays under *T20*, *T24* and *T28*, eggs were introduced at the start of the light phase of LD cycles. A red lamp (λ > 650 nm) was used for egg-collection, observation and fly handling under DD and during the dark phase of the LD cycles. The light phase of LD and LL was created with the help of a fluorescent white light of intensity ~100 lux (~0.15 W/m^2^). To estimate the egg-to-adult development time, vials with eggs were monitored daily for darkened pupae. Once the pupae became dark, vials were regularly monitored for freshly emerged adults. The number of males and females emerging every 2 h from each vial was counted. These 2 hourly checks were continued until no flies emerged from vials for the next 3 consecutive days. Pre-adult development time of a fly was calculated as the duration between the mid-point of 2 h egg-collection window and the mid-point of 2 h period during which the fly emerged as adult.

### Statistical analyses

Egg-to-adult developmental duration and durations of various pre-adult stages relative to the beginning of egg stage such as egg-to-hatching, egg-to-pupation and egg-to-wing pigmentation under various light regimes were analyzed separately using mixed-model analysis of variance (ANOVA) in which replicate populations (Block-B) were treated as random factor, light regimes (L) and stocks (S) as fixed factors crossed with populations. Post-hoc multiple comparisons were done using Tukey’s honestly significant difference (HSD) test. In all cases block average, i.e., average of the replicate vials in a population was used as the unit of analysis and hence, only the fixed factor could be tested for significance. All analyses were implemented on STATISTICA for Windows Release 5.0 B (StatSoft, 1995).

## Competing interests

The authors declare that they have no competing interests. The authors alone are responsible for the content and writing of the paper.

## Authors' contributions

PY and VKS conceived and designed the research. PY and MT performed the experiments. PY, MT and VKS performed the analyses. PY and VKS wrote the manuscript. All authors read and approved the final manuscript.

## Supplementary Material

Additional file 1: Figure S1 Shorter clock period in FD populations: Average free-running period (activity/rest rhythm and adult emergence rhythm) of selected (FD) and control (BD) stocks assayed under constant darkness (DD) at the 40th generation. For activity/rest rhythm a total of 32 adult flies per population (FD*1-4* and BD*1-4*) were recorded under DD at 25°C for a minimum of 10 cycles using Drosophila Activity Monitoring (DAM) system from Trikinetics, USA. The free-running period of the activity/rest rhythm was estimated using Lomb Scargle (LS) Periodogram in CLOCKLAB from Actimetrics, USA. The period of the replicate populations was estimated by averaging the period of individual flies. For adult emergence rhythm assay, approximately 300 eggs were dispenced into vials with 10 ml of banana medium kept under DD. Ten such vials per population were used in this assay. After the start of emergence vials were checked regularly every 2 h and the number of flies was recorded. Period calculation was performed by estimating the total duration for peak of emergence in every cycle. The error bars represents standard error around the mean (SEM). **Figure S2.** Spectral composition of white light source used during development time assay: Peaks of major components (different wavelength) of white light correspond to indigo (~420 nm), green (~570 nm), yellow (~590 nm), orange (620 nm) indicates their importance in the regulation of *Drosophila* development. Wavelength (in nanometer) is plotted along *x*-axis and *y*-axis represents light intensity (in arbitrary unit). The spectrum of white light was measured by a Hamamatsu mini spectrometer TM-series (C10083CAH). The final spectrum was obtained by averaging 100 spectra with accumulation time of 1 s each. Click here for file
