# Supplementary material for: Interaction of light regimes and circadian clocks modulate timing of pre-adult developmental events in Drosophila
Source: BMC Dev Biol. 2014 May 16;14:19. doi: 10.1186/1471-213X-14-19 (PMC4040135; doi:10.1186/1471-213X-14-19)
Supplement: Additional file 1: Figure S1 — Shorter clock period in FD populations: Average free-running period (activity/rest rhythm and adult emergence rhythm) of selected (FD) and control (BD) stocks assayed under constant darkness (DD) at the 40th generation. For activity/rest rhythm a total of 32 adult flies per population (FD1-4 and BD1-4) were recorded under DD at 25°C for a minimum of 10 cycles using Drosophila Activity Monitoring (DAM) system from Trikinetics, USA. The free-running period of the activity/rest rhythm was estimated using Lomb Scargle (LS) Periodogram in CLOCKLAB from Actimetrics, USA. The period of the replicate populations was estimated by averaging the period of individual flies. For adult emergence rhythm assay, approximately 300 eggs were dispenced into vials with 10 ml of banana medium kept under DD. Ten such vials per population were used in this assay. After the start of emergence vials were checked regularly every 2 h and the number of flies was recorded. Period calculation was performed by estimating the total duration for peak of emergence in every cycle. The error bars represents standard error around the mean (SEM). Figure S2. Spectral composition of white light source used during development time assay: Peaks of major components (different wavelength) of white light correspond to indigo (~420 nm), green (~570 nm), yellow (~590 nm), orange (620 nm) indicates their importance in the regulation of Drosophila development. Wavelength (in nanometer) is plotted along x-axis and y-axis represents light intensity (in arbitrary unit). The spectrum of white light was measured by a Hamamatsu mini spectrometer TM-series (C10083CAH). The final spectrum was obtained by averaging 100 spectra with accumulation time of 1 s each. [file 1471-213X-14-19-S1.pdf]

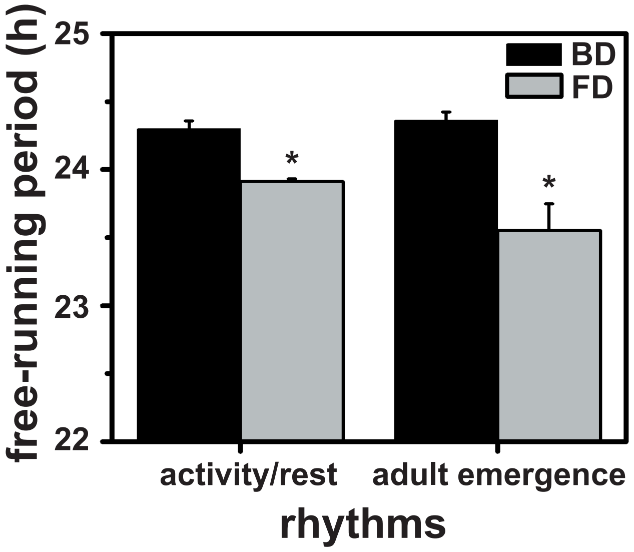

Figure S1. Shorter clock period in FD populations: Average free-running period (activity/rest rhythm and adult emergence rhythm) of selected (FD) and control (BD) stocks assayed under constant darkness (DD) at the 40th generation. For activity/rest rhythm a total of 32 adult flies per population (FD_{1-4} and BD_{1-4}) were recorded under DD at 25 ^{O}C for a minimum of 10 cycles using Drosophila Activity Monitoring (DAM) system from Trikinetics, USA. The free-running period of the activity/rest rhythm was estimated using Lomb Scargle (LS) Periodogram in CLOCKLAB from Actimetrics, USA. The period of the replicate populations was estimated by averaging the period of individual flies. For adult emergence rhythm assay, approximately 300 eggs were dispenced into vials with 10 ml of banana medium kept under DD. Ten such vials per population were used in this assay. After the start of emergence vials were checked regularly every 2 h and the number of flies was recorded. Period calculation was performed by estimating the total duration for peak of emergence in every cycle. The error bars represents standard error around the mean (SEM).

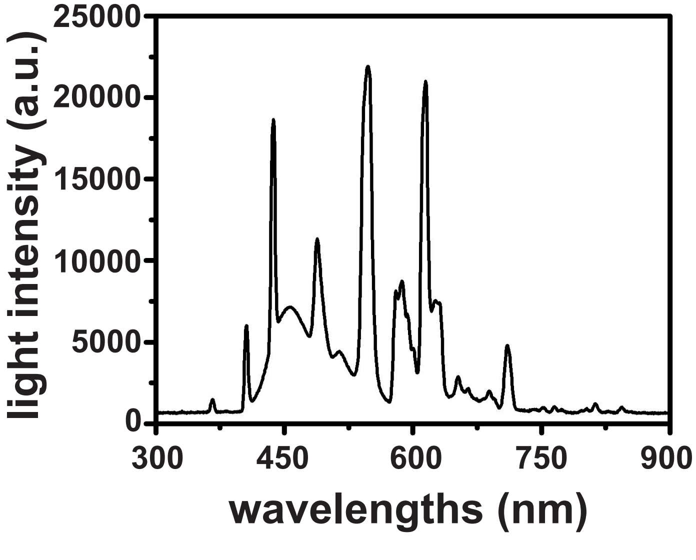

Figure S2. Spectral composition of white light source used during development time assay: Peaks of major components (different wavelength) of white light correspond to indigo (~420 nm), green (~570 nm), yellow (~590 nm), orange (620 nm) indicates their importance in the regulation of *Drosophila* development. Wavelength (in nanometer) is plotted along *x*-axis and *y*-axis represents light intensity (in arbitrary unit). The spectrum of white light was measured by a Hamamatsu mini spectrometer TM-series (C10083CAH). The final spectrum was obtained by averaging 100 spectra with accumulation time of 1 s each.
